# Interactions between motion and form processing in the human visual system

**DOI:** 10.3389/fncom.2013.00065

**Published:** 2013-05-20

**Authors:** George Mather, Andrea Pavan, Rosilari Bellacosa Marotti, Gianluca Campana, Clara Casco

**Affiliations:** ^1^School of Psychology, University of LincolnLincoln, UK; ^2^Institut für Psychologie, Universität RegensburgRegensburg, Germany; ^3^Department of General Psychology, University of PaduaPadua, Italy

**Keywords:** motion sensitivity, motion-streaks, motion perception, motion-form interactions, biological motion

## Abstract

The predominant view of motion and form processing in the human visual system assumes that these two attributes are handled by separate and independent modules. Motion processing involves filtering by direction-selective sensors, followed by integration to solve the aperture problem. Form processing involves filtering by orientation-selective and size-selective receptive fields, followed by integration to encode object shape. It has long been known that motion signals can influence form processing in the well-known Gestalt principle of common fate; texture elements which share a common motion property are grouped into a single contour or texture region. However, recent research in psychophysics and neuroscience indicates that the influence of form signals on motion processing is more extensive than previously thought. First, the salience and apparent direction of moving lines depends on how the local orientation and direction of motion combine to match the receptive field properties of motion-selective neurons. Second, orientation signals generated by “motion-streaks” influence motion processing; motion sensitivity, apparent direction and adaptation are affected by simultaneously present orientation signals. Third, form signals generated by human body shape influence biological motion processing, as revealed by studies using point-light motion stimuli. Thus, form-motion integration seems to occur at several different levels of cortical processing, from V1 to STS.

## Introduction

Anatomical and physiological studies of primates have identified over 50 distinct visual processing areas in the cerebral cortex (see Felleman and Van Essen, 1991). Ungerleider and Mishkin (1982) proposed that these multiple areas are organized into two major processing streams, known as the ventral stream and the dorsal stream, both originating in the primary visual cortex. This proposed division has since become widely established as a fundamental organizing principle in the primate visual system. The ventral stream travels into the temporal lobe, including cortical areas V4, TEO, and TE, and is thought to be crucial for the visual recognition of objects (also known as the “what” stream). The dorsal stream courses into the parietal cortex, and includes areas V3, MT, and MST, and is thought to be crucial for motion integration, for encoding spatial relationships between objects and for visual guidance toward objects (also known as the “where” stream). Single-unit recording studies are consistent with the two streams hypothesis. For example, neurons in areas forming part of the ventral stream show selectivity for color, shape and texture while those forming part of the dorsal stream show selectivity for the direction and speed of visual motion (see review in Maunsell and Newsome, 1987; Ungerleider and Pasternak, 2004).

The use of parallel streams to process different visual attributes has several merits (Marr, 1982). Modularity allows each stream to optimize its processing for the relevant visual attribute, rather than compromise for the sake of generality. For example, form processing is best served by high spatial acuity and low temporal acuity in order to code fine details reliably, while motion processing can sacrifice fine spatial acuity in favor of sensitivity to rapid temporal change. Moreover, modularity ensures that limitations or errors in processing output remain confined, rather than propagate across attributes.

However, in recent years evidence has accumulated which is inconsistent with the established principle of parallel, modular processing streams. The evidence reviewed in this paper demonstrates that form and motion are not processed independently in the visual system. On the contrary there is extensive interplay between form and motion processing systems which relies on a continuous exchange of information between different processing stages. The Gestalt psychologists, for example, recognized signs of this interaction long before the two streams hypothesis was proposed, when they formulated the principle of “common fate.” An invisible form composed of randomly arranged dots against a dotted background becomes immediately visible as soon as it moves, by virtue of the common fate of its dots, which all move together with a common speed and direction (see Uttal et al., 2000; see also Ledgeway and Hess, 2002 for similar results with motion-defined spatial contours; and Edwards, 2009 for motion-form interactions in common fate). This kind of figure-ground segregation shows clearly that forms can emerge from motion processing, in the absence of any other cue. The following sections review evidence for three other kinds of motion-form interaction, two at lower-levels of analysis and the other at higher-levels.

## Moving lines

One form of interaction between orientation signals and motion signals occurs in early visual areas. There is extensive physiological evidence that the receptive fields of direction selective neurons at low levels of analysis (V1) extract the motion component orthogonal to local orientation (Hubel and Wiesel, 1968; De Valois et al., 1982), so their directional response is ambiguous (the “aperture problem”). Neurons in extrastriate cortex (MT) solve the problem by integrating the responses of different V1 cells (Simoncelli and Heeger, 1998). Motion-selective cells in V1 respond more strongly to retinal motion in the direction perpendicular to their preferred orientation than to other directions. This response to the orthogonal motion component may explain a variety of perceptual phenomena. For example, the perceived speed of a line is more veridical when oriented orthogonally to its direction than when the line is tilted (Castet et al., 1993; Scott-Brown and Heeley, 2001). Furthermore, when bars slanted slightly away from vertical are oscillated up and down, the trajectories of the bars quickly become influenced by their orientation (Tse and Hsieh, 2007); the bars are perceived as moving up and down, but also at the same time sideways, creating the impression that the bars are following an elliptical trajectory.

The salience of a moving target line, i.e., the observers' ability to segment it from background noise lines, also depends on its orientation. Salience is increased when the orientation of the target line and its direction of motion are appropriately combined to match the property of the receptive field tuned to the orthogonal motion component. Indeed, when this component is available, the orientation of the line (Casco et al., 2006) and its motion direction are more easily discriminated. This has been shown for both two-frame (Casco et al., 2001) and multi-frame-motion sequences (Pavan et al., 2011). This last result in particular agrees with Nakayama et al.'s (1985) suggestion that spatial integration of motion signals is most efficient in a direction orthogonal to orientation. In multi-frame displays Pavan et al. (2011) showed that the consistent velocity of the orthogonal motion component in a target line allowed observers to detect it in the presence of the random frame-to-frame velocity and direction of noise lines. On the other hand, collinearity between target and noise does not aid detection (Alberti et al., 2010).

Thus, although there are end-stopped neurons in V1 that respond to the motion of line-terminators independently of line orientation (Pack et al., 2003), the orthogonal motion component is nevertheless important, and has been shown to affect the response of motion-selective neurons at later stages in MT (Pack and Born, 2001). The orthogonal component generates motion signals that may hinder the perception of veridical motion (Tse and Hsieh, 2007), but it can also improve motion segregation and grouping at very early stages of visual processing (Alberti et al., 2010; Pavan et al., 2011).

The end-stopped neurons reported by Pack et al. (2003) may also be implicated in another kind of motion-form interaction involving moving lines, in which the apparent direction of the lines is influenced by the shape of the aperture through which they are viewed. When an obliquely oriented drifting grating is presented behind an elongated horizontal aperture, the grating bars appear to move horizontally along the long axis of the aperture rather than obliquely, perpendicular to their own orientation (the “barberpole effect”). The bar or line terminators at the edge of the aperture appear to be particularly important for determining apparent direction (see Lorenceau and Shiffrar, 1992; Kooi, 1993; Fisher and Zanker, 2001; Badcock et al., 2003; Edwards et al., 2013). Terminators are created by the spatial form of the stimulus window. Psychophysical and neurophysiological evidence from aperture effects caused by terminators indicates that the underlying motion-form interaction takes place in a cortical area normally associated with the dorsal stream, namely MT (Pack et al., 2003, 2004).

In sum, research on moving lines reveals complex interactions between orientation and motion direction at the earliest levels of cortical analysis up to the point at which the aperture problem is solved, demonstrating that processing of these two attributes is inextricably linked.

## Motion-streaks

Sensory neurons cannot respond instantaneously to sudden, impulsive stimuli such as flashes of light. Instead their response builds up to a peak and then dissipates over a period ranging from tens to hundreds of milliseconds. For example, the response of retinal cone photoreceptors shows a peak ~70 ms after a bright flash and a trough at 150 ms (biphasic response); rod (monophasic) response peaks at 200 ms after the flash and does not return to baseline until a further 300 ms have elapsed (Schnapf and Baylor, 1987). Consequently, when a stimulus element translates rapidly across the retina, it leaves behind a trail of waning neural activity that is the likely neural substrate of “persistence of vision”; the motion-streaks seen behind bright moving objects such as fireworks. Persistence of vision can be viewed as an undesirable consequence of neural responses because of the motion blur it creates, and the biphasic temporal response of cones in bright light is arguably an attempt by the visual system to minimize its impact. But one obvious property of motion-streaks is potentially useful during motion processing: they are bound to be aligned with the axis of motion.

Cells tuned the orientation of motion-streaks should be maximally activated by them. Thus, rapid retinal motion produces responses both in motion-selective cells tuned to that direction, and in orientation-selective cells tuned to an orientation aligned with the axis of the motion—the motion-streak. Psychophysical evidence from orientation detection and after-effects, as well as recent neuroimaging data, is consistent with the view that motion-streaks excite orientation-tuned cells in the human visual system (Alais et al., 2011; Apthorp et al., 2011, 2013). Geisler (1999) proposed that the outputs of motion- and orientation-selective cells are combined in visual cortex to create a “spatial motion-direction” (SMD) sensor tuned to both streak orientation and motion direction. He also presented psychophysical evidence for the existence of such sensors. Luminance detection thresholds were measured for moving Gaussian blobs, in the presence of dynamic random line masks oriented either parallel or orthogonal to the axis of motion. Mask orientation had no effect on thresholds at low blob speeds, but above a critical speed, parallel masks elevated detection thresholds relative to orthogonal masks, consistent with the SMD sensor. A limitation of this experiment is that it did not specifically measure motion discrimination, but instead employed a 2AFC detection paradigm. So one cannot be sure that the masking effect revealed anything about motion perception.

Ross et al. (2000) generated static random Glass patterns by taking a field of randomly positioned dots, and giving each dot a partner displaced from it by a short distance corresponding to rotation of the original dot about the center of the pattern by a fixed angle. When a series of such uncorrelated patterns is presented rapidly, observers report apparent rotation even though there is no dot-to-dot correspondence between successive patterns. Ross et al. (2000) interpret this effect as consistent with Geisler's (1999) SMD sensor (see also Krekelberg et al., 2005 for similar results). Burr and Ross (2002) addressed the limitation of Geisler's original threshold study by employing a task that required observers to discriminate the direction of motion. Thresholds were higher for random line masks parallel to motion direction than for masks perpendicular to motion direction, consistent with Geisler's findings. Edwards and Crane (2007) further provided evidence of a motion-streak mechanism using a 3-frame global-motion stimulus and manipulating the strength of the motion-streak. When the same dots carried the global-motion signal over successive motion frames (long-streak condition) lower thresholds were obtained at high speeds (consistent with a motion-streak system). This facilitation decreased markedly at low contrast, due to reduced motion-streak magnitude and to the low contrast sensitivity of form cells contributing to motion-streak extraction. In addition to their effect on motion thresholds, motion-streaks also alter the appearance of supra-threshold motion. Several papers report changes in the apparent direction of moving elements when they are superimposed on a static background of tilted lines (see Swanston, 1984; Khuu, 2012). A possible mechanism for this direction effect involves mutual inhibition between orientation-selective cells, some of which are activated by the tilted background while others are activated by the motion-streak. The resulting angle-expansion effect propagates to the motion system via the SMD sensor.

On the basis of the research surveyed so far, it cannot be argued that form and motion are processed by completely independent systems. Evidence indicates that the interactions between orientation signals and motion signals are likely to occur in early visual areas (e.g., V1, V2).

Claims for motion-form interactions beyond V1/V2 cannot be based simply on evidence for long-range interactions, since these can occur in V1 as contextual modulation of responses (Alexander and van Leeuwen, 2010). Instead they should relate to effects associated with the specific functions performed by higher-level cortical areas. Area MT is believed to be involved in the integration of directional motion signals. For example, adaptation to two superimposed fields of dots moving in different directions normally produces a unidirectional motion after-effect (MAE) in the direction opposite to the vector average of the adapting directions (Mather, 1980; Verstraten et al., 1994; van der Smagt et al., 1999; Verstraten et al., 1999; von Grünau, 2002; Alais et al., 2005), and the integration of the two adapting motion components is thought to occur in extrastriate cortex in the dorsal stream, most likely in area MT as mentioned earlier. Mather et al. (2012) psychophysically investigated motion-form interactions at this integration stage of processing. Their results showed that superimposing a static grating orthogonal to the direction of the resultant unidirectional MAE during adaptation reduced the strength of the MAE relative to a condition in which the grating was parallel to the resultant MAE direction. Thus, the strength of bi-directional motion adaptation was modulated by simultaneously presented orientation signals. These findings provide evidence that form and motion signals interact at the global motion level where moving components are integrated, i.e., at a level of processing which is clearly part of the two-stream architecture.

Neurons in area MST of the dorsal stream are closely associated with the analysis of global patterns of motion (i.e., optic flow; Graziano et al., 1994). Neurons in the dorsal part of area MST (i.e., MSTd) of the macaque have large receptive fields (from 10° up to 100°; Desimone and Ungerleider, 1986; Tanaka and Saito, 1989) and show selectivity to optic flow and to its components (Sakata et al., 1985, 1986; Saito et al., 1986; Tanaka et al., 1986, 1989; Tanaka and Saito, 1989; Duffy and Wurtz, 1991b; Lagae et al., 1993; Graziano et al., 1994). There is psychophysical evidence for motion adaptation at the level of optic flow analysis, in the form of the phantom MAE. In phantom MAEs, adaptation of some parts/sectors of the visual field to complex motion components such as expansion (or contraction) induces the perception of contraction (or expansion) in other (non-adapted) parts of the visual field during testing. The phantom MAE is likely to reflect adaptation of cells with large complex receptive fields at the level of MST (Regan and Beverly, 1985; Desimone and Ungerleider, 1986; Tanaka and Saito, 1989; Duffy and Wurtz, 1991a; Lagae et al., 1993; Graziano et al., 1994; Morrone et al., 1995; Snowden and Milne, 1996, 1997; Burr et al., 1998). Pavan et al. (2013) used the phantom MAE to test for the presence of form-motion interactions at this high-level site of adaptation in the dorsal stream. Their results showed that adding a concentric grating orthogonal to radial optic flow during adaptation suppressed the duration of the phantom MAE, compared to a radial grating parallel to the global pattern of motion. This may indicate an interaction between form and motion signals at the level in which optic flow is processed.

Recent evidence indicates that inferences about stimulus selectivity based on an adaptation paradigm are not necessarily straightforward (Rentzeperis et al., 2012). Nevertheless, in the case of motion-form interactions during optic flow analysis, evidence from Niehorster et al.'s (2010) discrimination study bears out Pavan et al.'s (2013) adaptation study. Niehorster et al. (2010) showed that human heading perception in a heading direction discrimination task was based on a combination of motion (optic flow component) and form (radial glass patterns) signals. There is evidence for neurons in the form processing stream which are sensitive to these radial streak patterns (Gallant et al., 1993; Ostwald et al., 2008). The visual system may take advantage of the close correspondence between visual form and motion signals generated by locomotion, combining the two during high-level optic flow processing.

## Biological motion

Johansson (1973) introduced highly impoverished “point-light walker” movies in which moving human figures are visible only by means of isolated points of light fixed at the major joints (ankles, knees, hips, wrists, elbows, shoulders). Naive observers are able rapidly and reliably to perceive many human attributes in these movies despite the paucity of available information, including the actor's gender, mood, and action type (see review in Blake and Shiffrar, 2007). Point-light walker displays are now also widely known as biological motion displays. In the forty years since their introduction biological motion displays have attracted debate and dispute regarding the neural processes which mediate their perception; do they involve form analysis (the ventral stream), or motion analysis (the dorsal stream), or both? At first sight one might think that biological motion displays specifically target motion analysing processes, since there are no explicit visual connections between any of the dots. Indeed many psychophysical studies attest to the importance of motion signals. Spatiotemporal properties such as display duration, dot displacement distance and inter-frame interval are all critical to biological motion perception, consistent with a reliance on information in the dorsal stream (e.g., Johansson, 1976; Mather et al., 1992; Thornton et al., 1998). However, low-pass spatial filtering of any single frame in a biological motion sequence would reveal a blurred, body-shaped form which could serve as a stimulus for form processing. A number of spatial properties do affect biological motion perception in a way that implicates processes in the ventral stream. Beintema and colleagues limited the display lifetime of individual dots (Beintema et al., 2006) or shifted dots around the body on a frame-by-frame basis so that they were not placed consistently at the joints (Beintema and Lappe, 2002), and at least some degree of biological motion perception survived both manipulations. Thus, it is difficult to argue against the proposition that biological motion analysis involves both the dorsal *and* ventral streams. The question then arises as to where in the cortex is the information from the two streams combined. Regions within the rostral Superior Temporal Sulcus (STS) receive information from both streams, so STS is a likely area of convergence (Ungerleider and Pasternak, 2004). Geise and colleagues have developed and tested a computational model of biological motion analysis that conforms to this architecture: separate analyses in the dorsal and ventral streams converge on a common representation in high-level areas such as STS (Giese and Poggio, 2003; Fleischer and Giese, 2012). Neuroimaging data is consistent with this hierarchy, and also implicates extrastriate and fusiform body areas (EBA and FBA; Jastorff and Orban, 2009). Fleischer and Giese (2012) acknowledge, however, that segregation of signals until they reach very late stages of cortical analysis may be an oversimplification. Many studies use background “noise” dots to mask form-based cues, either moving in a random fashion or in a way that mimics the local motion of the figure dots. The presumption is that noise dots abolish form cues, since the form is invisible in each frame. However, given the well-known common fate Gestalt principle described in the Introduction, one could argue in favor of a low-level inter-play between form and motion processing in which motion-mediated common fate allows the visual system to segregate dots representing the body form from the background dots, and later motion and form processes extract gender, mood and so on.

Form processing of biological motion in Giese and Poggio's (2003) model includes “snap-shot” neurons which are selective for specific body shapes that are adopted during movement. The output of these ventral stream neurons allows motion to be inferred from body shape. As Giese and Poggio (2003) state (p. 184) “active snapshot neurons pre-excite neurons that encode temporally subsequent configurations, and inhibit neurons that encode other configurations.” Lange and Lappe's (2006) form-based model of biological motion analysis employs similar posture-specific form cells to encode the different body configurations adopted while walking, and a coding scheme based on their sequential activation.

Artists have traditionally been able to convey an impression of motion in static artworks such as painting and sculpture using poses which imply motion because they would be physically impossible for a human actor to hold for any length of time. Vision scientists call such static depictions of action “implied motion.” The snap-shot or posture-specific neurons in the ventral stream proposed by Giese and Poggio (2003) and Lange and Lappe (2006) are a plausible neural substrate for the encoding of implied motion. There is accumulating evidence that activity originating in such neurons finds it way to cells in the dorsal stream. Senior et al. (2000) used fMRI to identify brain regions activated by video clips of objects in motion, and clips of the same objects at rest. Activation in dorsal area MT was higher while participants viewed the movie clips, as one would expect for an area involved in motion analysis. Interestingly, Senior et al. (2000) also found higher activation in MT while participants viewed still images implying motion, compared to images containing no implied motion. Similar results were reported by Kourtzi and Kanwisher (2000). A plausible source of MT activation by implied motion is cells in the dorsal stream that are sensitive to the motion implied by form; snap-shot neurons. Alternatively, recent neuroimaging results indicate that cells sensitive to static body shape and to motion are actually intermingled in area MT (Ferri et al., 2013). Thus, the interaction between the form and motion pathways may not be confined to convergence at the level of STS, but could involve cross-activation at the level of MT. Winawer et al. (2008) exposed experimental participants to rapidly presented sequences of unrelated static images each containing implied motion, and reported that this “adaptation” generated a motion aftereffect on a directionally ambiguous dynamic test pattern (see also Pavan et al., 2011; Pavan and Baggio, 2013, for similar results). Such results would be consistent with cross-activation of MT by neurons in the dorsal stream, because MT neurons have long been associated with motion adaptation. However, Morgan et al. (2012) sound a note of caution, arguing that the post-adaptation directional bias found by Winawer et al. (2008) could be due to a shift in decision bias rather than a shift in the relative activity of direction-selective neurons. Unlike Winawer et al. (2008), Pavan et al. (2011) employed a control adapting condition that did not contain implied motion, but still allowed the possibility of response bias. They did not obtain an after-effect in this condition.

## Summary

Visual motion and form information is inextricably linked in the sense that motion is, by definition, spatiotemporal; change over both time *and* space. The research reviewed here indicates that the two components of motion interact at multiple levels of processing. Prior to segregation into parallel dorsal and ventral streams, the salience and apparent direction of moving lines depends jointly on line orientation and motion. The SMD sensor is designed to exploit the orientation signals generated by fast motion in the form of motion-streaks. Evidence from research on implied motion and biological motion indicates that interactions between form and motion processes also occur after the point at which the dorsal and ventral streams diverge, probably in area MT, as well as at the point of convergence in STS.

Thus, the visual system seems to take advantage both of modular processing and data sharing, by allowing data to flow between specialized neural processing streams. The theoretical justification for these interactions rests on the high degree of correlation between the signals in different modules, due to their common origin in natural images. Integration of signals across processing modules serves to minimize signal redundancy and maximize signal reliability.

### Conflict of interest statement

The authors declare that the research was conducted in the absence of any commercial or financial relationships that could be construed as a potential conflict of interest.
